# A Call to Journal: Grief Work and Poetry—A Reflection

**DOI:** 10.1177/15423050231219748

**Published:** 2023-12-14

**Authors:** Janet L. Kuhnke

**Affiliations:** School of Nursing, 55964 Cape Breton University, Sydney, Nova Scotia, Canada

**Keywords:** Grief, journaling, spiritual care

## Abstract

In this poetic work, journaling is shared as a means to navigate grief. The call is to spiritual leaders to encourage those grieving to be creative in their journey. This is a tool to support understanding the experience of grief as a healthcare provider.

## Introduction

The grief experienced is an individual journey. How one lives in the space of grief and loss is unique to each person. In this reflective work, I share examples of poetic writing that supported my growth and understanding of personal grief (Mooney, 2022). This is important work as it is intended to support spiritual care leaders such as pastors, chaplains, clinical counselors, etc., in their practice alongside families and health care providers (Sergi & Muller, 2022). Corpuz (2023) eloquently states “death is not a problem to be solved but a reality” (p. 74). Grief can be explored in efforts to understand its complexity.

In this case, the grief experienced was a catalyst to journal about the loss of a loved one. I wanted to understand the deep sadness and sense of heartache and pain in which I was experiencing (Lundquist & Husebo, 2019). Journaling my story brought comfort, it was a place where I could quietly perform far away from day-to-day living (Fels, 1998). I often sat and wrote reflectively about how I thought, felt and reflected upon memories of my dear loved one. It was my way of creating art, words pouring forth, from which I tried to draw comfort. In my grief, my time writing was a place where I could dance with loss as it moved across my landscape.

Writing can be understood as a frame from which one may reflectively grow and gain insight. I wanted to understand grief, not as an academic, but as a daughter. I wanted to stand alongside grief and not collapse under its shroud and ambiguity (Montgomery, 1946). This was an important process as I could not find a road map for how to be, feel and live (Earle, 2006). Yet, I also knew in life, I could “wear grief as a secret charm, where none may see-close to the heart of me!” (Montgomery, 1946, p. 109). I knew only my journaling might disclose my innermost being, my pain and loss. For me, grieving was additionally challenging as we were immersed in the nationwide response to the coronavirus pandemic limiting my ability to participate in family and church grief ceremonies (Eden et al., 2022; Roman et al., 2020).

I also sought to demystify the role of writing within the grief experience (Boss, 2010). Self-understanding and personal growth are needed in order to support others, and to be healthy when in-service (David et al., 2022). I sought comfort in journaling, as I understood writing as a therapeutic tool for healthcare providers to utilize (Wurtz et al., 2022). I also wanted to move the activity of journaling past the stricter role of documenting and monitoring one's health functions (e.g., keeping track of one's blood pressure) to a person-centered way of being where I could grow in self-understanding and insight into my emotions (Alexander et al., 2016) and spiritual wellness and faith (Fish & Shelly, 1978). In turn, as a spiritually based nurse I knew that grieving was not where I would choose to be, yet, I have learned that I am in this state because that is where God wants me (Lockerbie, 1964).

In literature, journaling is understood to support health and bring forth emotional and spiritual wellness (Ansari, 2022), especially in the loss of a parent (Spear, 2005). Journaling is widely recognized as an opportunity to share one's innermost thoughts, insights, and glimpses into circumstances throughout life (Alexander et al., 2016; Earle, 2006; Kozoil, 2021). As a poetic form, journaling may also support spiritual growth (Kuhnke, 2022) and mental health and wellness (Kuhnke, 2021). Wright (2004) reminds us that the act of writing about grief may encourage healing and the releasing of energy when living seems impossible. In grief, it was spontaneous journaling in which I found myself immersed (Harvey, 2011). It was in this place where I found a space to express emotions through poems rich, in description of grief. Therefore, four short poems are shared from my private journals written after the parting of my loved one. I hope that spiritual care leaders (pastors, chaplains, clinical counselors, etc.) will encourage others and themselves to journal through poems as a step to growth through grief and loss. The following poems were inspired by readings including Alexander's hymn book (1915), a devotional book gifted to me by a friend (Lockerbie, 1964), and writings on grief (Wright, 2004).

## In My Garden

I wanted to talk to you today.
I was teary and intensely missing the ability
To just pick up the phone, and call.
I was kneeling, digging holes for sunflower seeds.
Tears drowning my being.
The fragrance of the purple lilacs, heady in the afternoon sun wrap around me
Immerse me in quietness.
The fragrance reminds me of the lilacs outside my window as a child.
They remind me of your love of growing plants and
The endless efforts to order, plan, seed, and water the growing plants.
You were always there Dad, at the end of the phone.
You would adjust your hearing aid,
And were willing to listen, counsel and
Remind me of my faith.
You are so missed.
Some comfort prevails as I dig,
In the earth warmed by the sun.
I clench the soil,
Shrouded and
Yearning to be with you.
Another season, the earth presents around me.
I know my faith will carry me,
Holding me steady, piloting me. (*Journal entry*, July 2020)

## Can I Call You Today?

It is Christmas time, and I wanted to talk to you today.
I wanted to call and tell you about my latest creative ventures.
I wanted to tell you a funny story about how I messed-up 
in life.
You would have answered the phone and
We would have chatted for an hour or so, and
Laughed.
You would have told me about your ventures and building 
projects.
We would have chatted about the weather,
The harvesting season and corn production levels, and
The next winter storm pending.
You would have given me an update on the family and
Your long-time friends at church.
You would remind me to pray for leaders and those in charge,
It is in these times,
I miss you deeply. (*Journal entry,* December 2020).

## Snow Globe and Tears

It is Christmas time, my second season without you.
I am in my workshop, painting colorful scenes to brighten my soul.
I am comforted by the earth swirling in snow,
Like a fragile snow globe from my childhood.
To the north, the snow falls thickly across the mountain.
To the east, it whips into dancing Irish fairies,
Twirling and swaying across the land
Driven by the winds.
To the south, the fir trees wave wildly.
Dropping snow from their branches
Quietly to the earth—eerily, soothing my soul.
To the west, the tail of the red fox disappears along the tree line,
Deep among the apple trees
Hunting for dinner, the next catch.
You are missed today and every day.
I do not understand what they call grief, but
I am trying to stand alongside it, not to be buried.
I am shrouded and comforted by
Creative works, colours, and varied art mediums.
Books about grief, on my lap
With notes in the margins, seeking truth.
Colorful bookmarks call me onward to the
Next page, to read more.
Apparently, this flood of tears will pass and
These waves of emotion are okay. (Journal entry, January 2021)

## Standing, Grief Is All Around Me

Today is today,
In the moment—it is now.
In this very moment, and in many throughout the day,
I miss you.
This morning
I am singing from one of your old hymn books
Tears pour, tissue ready at my side.
I walk to my art shop to create and
Distract myself.
I stand and sob quietly, you would have loved this space.
We would have talked about different types of wood and how to harvest each.
I remember you taking wood-working courses.
I remember your wood lathe and you showing me how to use the tools.
Gluing wood together
It seemed to take forever,
Before the wooden block was ready for use.
Then you moved it into position
On the lathe, and
Creations emerged.
I have one of your wooden serving plates and a beautiful wooden bowl with a lid,
Made from the maple wood, milled from the farm.
Dad, they remind me of your work ethic and diligence to learn and
Your kindness in teaching.
They are lovely and
I still miss you.
In response, I left my shop,
Praying for solace and comfort.
I hiked into the bush and across the land.
The cold winds briskly rush the morning clouds across the skies,
Racing to where, heaven?
The rose-red hues light up the early morning skies,
Bringing bright hues of hope.
Quietness surrounds my heart and soul,
Yet, I dance with grief, and
Grief dances with me. (*Journal entry*, November 2021)

## Discussion

The preceding poems are purposefully shared as encouragement to others experiencing grief and loss. Also, they are intended to support spiritual care advisors who encourage persons to journal and write about their experiences (Spear, 2005). When I experienced the loss of a parent I was not prepared for the ensuing feelings and emotions. Caplan et al. (2005) state that as we age, we shift from focusing on how the loss occurred, to what new meaning can be gleaned from the experience. They state we also shift from what was, to what may become, especially in relation to our faith.

In time, I too moved from wondering “what the dark shroud was around me?” I moved onward to new reflections, insights, and possibilities in life, without my dad. I truly was surprised by grief, not sure of the sensation of being wrapped tightly in a blanket, to wondering if I would recover from its embrace (Lewis, 1961). Though as a nurse, I analytically knew death was normal and part of life, I was not prepared for the enduring sadness that prevailed (Dean, 2011). Journaling, telling my story, hopefully a teaching story (Coles, 2004), interrupted the threads of pain, woven thickly into a blanket. Writing was and is my ongoing attempt to move grief into a place where I can learn from it and become wiser.

Finally, I know that no two journeys of grief and loss are the same (Wright, 2004). The circles of life continue from birth to life—a living time—and now ending time. It was during this ending time that writing poems in journals supported a coming to understand more fully the emotions and experiences of grief (Mooney, 2022). Grief is real. I came to understand that I can stand alongside grief and feel associated emotions. Journaling was a place and space to grow and gain comfort. In my writing, I lived out my grief and continue to find some solace in writing about emotions related to grief.
